# Effect of Starch Plasticization on Morphological, Mechanical, Crystalline, Thermal, and Optical Behavior of Poly(butylene adipate-co-terephthalate)/Thermoplastic Starch Composite Films

**DOI:** 10.3390/polym16030326

**Published:** 2024-01-25

**Authors:** Xiaoyan He, Fuhong Zhang, Congcong Li, Weiwei Ding, Yuanyuan Jin, Lisheng Tang, Ran Huang

**Affiliations:** 1 Department of Material Science and Engineering, Taizhou Institute of Zhejiang University, Taizhou 318000, China; 2 Sanmen Megatron Tech. Co., Ltd., Taizhou 318000, China; 3 Center for Biotechnology and Biomedical Engineering, Yiwu Research Institute of Fudan University, Yiwu 322000, China; licongcong@ywfudan.cn; 4 Academy for Engineering and Applied Technology, Fudan University, Shanghai 200433, China

**Keywords:** PBAT, plasticized starch, biodegradable films, optical property

## Abstract

Starches plasticized with glycerol/citric acid/stearic acid and tributyl 2-acetylcitrate (ATBC), respectively, were processed with poly (butylene adipate-Co-terephthalate (PBAT) via extrusion and a film-blown process. All the composite films were determined for morphology, mechanical, thermal stability, crystalline, and optical properties. Results show that the most improved morphology was in the 30% glycerol plasticized PBAT/thermoplastic starch (TPS) composite films, characterized by the smallest and narrowest distribution of TPS particle sizes and a more uniform dispersion of TPS particles. However, the water absorption of PBAT/TPS composite films plasticized with glycerol surpassed that observed with ATBC as a plasticizer. Mechanical properties indicated insufficient plasticization of the starch crystal structure when using 10% ATBC, 20% ATBC, and 20% glycerol as plasticizers, leading to poor compatibility between PBAT and TPS. This resulted in stress concentration points under external forces, adversely affecting the mechanical properties of the composites. All PBAT/TPS composite films exhibited a negative impact on the initial thermal decomposition temperature compared to PBAT. Additionally, the haze value of PBAT/TPS composite films exceeded 96%, while pure PBAT had a haze value of 47.42%. Films plasticized with 10% ATBC, 20% ATBC, and 20% glycerol displayed lower transmittance values in the visible light region. The increased transmittance of films plasticized with 30% glycerol further demonstrated their superior plasticizing effect compared to other PBAT/TPS composite films. This study provides a simple and feasible method for preparing low-cost PBAT composites, and their extensions are expected to further replace general-purpose plastics in daily applications.

## 1. Introduction

Poly (butylene adipate-co-terephthalate) (PBAT) has attracted great interest as a potential substitute for low-density polyethylene (LDPE), particularly in packaging applications such as lawn waste bags, food containers, or film wraps [1]. PBAT is particularly desirable due to its biodegradability, excellent flexibility, thermal stability, and outstanding water barrier properties [2,3]. Moreover, as an aliphatic-aromatic co-polyester, PBAT combines the advantageous mechanical properties of aromatic polyesters with the biodegradability expected of aliphatic polyesters [4,5]. Despite these merits, PBAT is seldom used alone in food packaging due to its inadequate oxygen barrier properties and high cost [6,7].

Among natural polymers, starch has emerged as a promising option for food packaging, given its biodegradability, film-forming properties, abundance, low cost, and good oxygen barrier properties [8]. However, starch alone lacks the mechanical strength and thermal stability required for effective polymer use. Native starch has to be modified in order to be melt-processed as a thermoplastic material because the melting temperature (Tm) of pure dry starch is close to 220–240 °C, which is higher than its degradation temperature (220 °C). Gelatinization of starch at elevated temperatures, typically in the presence of a plasticizer such as water or glycerol, is a common approach to producing thermoplastic starch (TPS). This process significantly enhances the flexibility and processability of the material [9]. Although products made from TPS exhibit good oxygen barrier properties, biodegradability, and compostability, some deficiencies, such as strong water absorption, relatively poor mechanical properties, and low thermal stability, limit their uses [10,11,12,13]. Furthermore, products made from TPS swell and deform upon exposure to high-moisture media. To alleviate these deficiencies, several strategies have been used, including melt blending of TPS with other conventional or biodegradable polymers. PBAT, with high flexibility and stability in the extrusion process, becomes an ideal complement. Therefore, blending TPS with PBAT facilitates film production and achieves the desired flexibility for wrapping and sealing processes.

However, with the usage of starch materials, small molecular plasticizers will progressively migrate out, which is starch retrogradation, resulting in a reduction in the quality of the materials [14,15]. In recent years, researchers have explored various plasticizers and coupling agents to modify PBAT/starch composites. Dammak et al. investigated the effect of maleated PBAT (PBATg-MA) and maleic anhydride (MA), citric acid (CA) coupling agents on the mechanical properties and morphology of PBAT/TPS composites, In the absence of the compatibilizer and in the presence of PBAT_g_-MA, the PBAT was the continuous phase, while the TPS became the continuous one in the presence of MA and CA. These evolutions were explained by the change in the melt rheological properties of TPS in the presence of the compatibilizer and its aptitude to promote interfacial adhesion between TPS and PBAT phases [1]. Zhang et al. found that tartaric acid (TA) plays diverse roles in PBAT/TPS-TA composites [16]. TA primarily serves as an acid catalyst, reducing both the molecular weight of starch and the shear viscosity of TPS. As a result, TPS-TA particles can disperse uniformly in the PBAT matrix without agglomeration. When TA contents are below 1%, another crucial role of TA is the coupling effect, enhancing compatibility between TPS and PBAT. However, it also decreases the interface interaction between TPS and PBAT, leading to a phase morphology reversal in PBAT/TPS-TA-4 from a homogeneous phase to a “sea-island” structure. Li et al. combined CS particles (native starch and mechanically activated starch) with ATBC and PBS together through melt blending. The impact strength of composites was increased by 126%, and the elongation at break increased by more than five times compared to PBS, showing a ductile fracture trend. Moreover, the water absorption test and contact angle test demonstrate a significant improvement in water resistance [17].

To sum up, the synergistic plasticization of starch with small molecular compatibilizers, combined with glycerol-plasticized starch, provides a viable solution for the preparation of TPS. ATBC demonstrates the potential to plasticize starch, imparting improved ductile properties to PBS composites. No published studies have conducted comparisons between the effects of glycerol and ATBC as plasticizers on the properties of PBAT/TPS films. Recognizing the promising complementary properties between TPS and PBAT composites, this study focuses on the preparation of TPS using two different primary plasticizers. The physical properties of the PBAT/TPS mixtures are compared, in particular, TPS plasticized with glycerol/citric acid/stearic acid and with ATBC. The objective is to assess their effectiveness and examine how they influence the properties of the PBAT/TPS composite.

## 2. Materials and Methods

### 2.1. Materials

Poly (butylene adipate-co-terephthalate) (PBAT, MFR 4–6 g/10 min at 190 °C) was purchased from Xinjiang Blue Ridge Tunhe Chemical Industry Co., Ltd., Changji, China. Native corn starch containing 73% amylopectin and 27% amylose (purity 99%) was provided by Binzhou Jinhui Corn Development Co., Ltd., Binzhou, China. Stearic acid was obtained from Oledchemicals Industry, Kuala Lumpur, Malaysia. Citric acid (purity 99.5%), glycerol (purity 99.5%), tributyl 2-acetylcitrate (ATBC, purity 97%), and all other chemicals were supplied by Macklin Chemical Co., Ltd., Shanghai, China.

### 2.2. Gelatinisation of Starch

The corn starch was dried at 90 °C in the oven for 48 h to make the moisture content lower than 0.1% and then mixed with glycerol, citric acid, and stearic acid in a high-speed mixer at the mass ratio of 100:20:0.6:0.3 and 100:30:0.6:0.3 for 10 min, respectively. The temperature of the mixer was cooled and kept at 30 °C during the mixing process.

Two doses of corn starch plasticized by ATBC were prepared by adding ATBC at mass ratios of 100:10 and 100:20 and stirring thoroughly. Subsequently, all the mixtures were kept in a closed vessel to avoid absorption of moisture and were kept for 24 h before processing with an extruder.

### 2.3. Preparation of PBAT/TPS Composite Films

The melt mixing of PBAT/TPS biodegradable composites was carried out in a co-rotating twin screw extruder (KYmach SJ-30, Nanjing, China), with a variable temperature difference of 90–140 °C from the feed to the die zone, and a screw speed of 300 rpm. PBAT and TPS were compounded at a ratio of 70:30. The drawing rods were then cooled, pelletized, and stored in a sealed package until further use.

PBAT/TPS blended films were prepared by film blown using a single screw extruder with a screw diameter of 170 mm and an L/D ratio of 30 (Leibo, Hangzhou, China). The film-blown temperature was independently controlled at four zones along the extruder barrel, and a strand die was used to achieve a temperature profile in the range of 135–150 °C. The screw speed was set at 30 rpm, and the feed rate was 12 rpm. Films were named PBAT, PBAT/TPS-10% ATBC, PBAT/TPS-20% ATBC, PBAT/TPS-20% glycerol, and PBAT/TPS-30% glycerol. The thicknesses of PBAT/TPS composite films were 65 ± 5 μm.

### 2.4. Characterization of PBAT/TPS Composite Films

The surface morphology of PBAT/TPS composite films was examined using an optical microscope (OLYMPUS CX31, Church Hill, TN, USA). Images were taken at brightfield, transmission mode, with a magnification of 4–10×.

For each composition, five pieces of film were cut into 20 mm × 20 mm and stored at 55% RH for 7 days before testing and then dried in the oven at 105 °C for 24 h. The water absorption test was determined by immersing oven-dried samples into distilled water at room temperature. The weight of the samples was monitored and recorded at regular intervals (2 h, 4 h, 6 h, 8 h, 24 h, and 48 h). The water absorption ratio was calculated using Equation (1).

(1) $$Yield\;\left(\%\right)=\frac{M_{t}-M_{i}}{M_{i}}\times 100$$

where *M_i_* is the initial mass of the sample, and *M_t_* is the mass after water absorption.

The X-ray diffraction (XRD) was used to conduct the crystallinity of samples. The samples were analyzed in the X-ray diffractometer (Model: Rigaku Iniflex 600, Tokyo, Japan) using Cu-Kα radiation (λ = 0.154 nm) at 40 kV and 30 mA with a goniometer speed of 0.02 s^−1^. The spectra were measured for 2θ in the range of 5–50°. The X-ray detector used was a scintillation counter with a detector angle of 40°, placed at a distance of 300 mm.

The mechanical properties of films were meticulously assessed through traction tests, according to the ASTM Standard method D882-12 (ASTM, 2012) [18]. Rectangular films with a width of 15 mm and a length of 150 mm were carefully prepared and positioned in an electronic tensile testing instrument with a charge cell of 500 N. The samples were then subjected to a controlled stretching rate of 300 mm/min, with an initial gauge distance of 50 mm. Samples were conditioned at 23 °C and 50% relative humidity for 72 h before testing. Six specimens were tested for each composition.

Thermal weight loss analysis was conducted using a thermogravimetric instrument (TGA 2, Mettler Toledo, Zurich, Switzerland). In this analysis, approximately 8–10 mg of the polymeric materials were placed in an aluminum dish under the nitrogen (N_2_) atmosphere at room temperature. The sample was then subjected to heating at a rate of 10 °C/min using a high-resolution dynamic mode until it reached a temperature of 600 °C. Throughout the heating process, the weight loss of the sample in response to the temperature change was recorded.

The haze properties of the films were measured using an optical tester (YH1200, 3NH, Shenzhen, China), and different positions across the film were assessed for both transmittance (T) and haze (H).

The color of the different samples was determined using a color spectrophotometer (Leeuwarden, The Netherlands). A spectrophotometer was used to measure the color of the sample obtained. This apparatus measures the color in the CIE Lab (L*, a*, b*) scale. Each set of samples underwent three individual measurements, and the resulting average value was recorded.

The UV and visible light transmittance of the films was determined using a U-4100 UV–vis spectrophotometer (HITACHI, Japan), operating in the range of 300–800 cm^−1^ and with a scan speed of 300 nm/min.

## 3. Results and Discussion

### 3.1. Surface Morphology of Films

**Figure 1:**
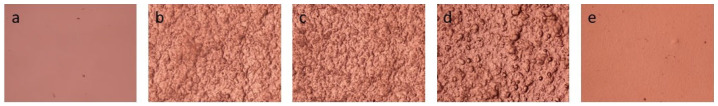
Surface morphology of PBAT/TPS films; glycerol/citric acid/stearic acid and ATBC serve as the plasticizers of TPS, respectively, PBAT (**a**), PBAT/TPS-10%ATBC (**b**), PBAT/TPS-20%ATBC (**c**), PBAT/TPS-20% glycerol (**d**), PBAT/TPS-30% glycerol (**e**).

Figure 1 illustrates the surface morphology of neat PBAT film and PBAT/TPS composite films. The surface of neat PBAT film appeared smoother and more uniform than that of the 10% ATBC, 20% ATBC, and 20% glycerol plasticized films. When 10% ATBC and 20% ATBC served as the plasticizer, PBAT/TPS films (Figure 1b,c) exhibited rough and uneven features; the size of the phases and the lack of adhesion between the two phases evidenced immiscibility. Figure 1d shows the typical sea-island morphology of uncompatibilized composites; it can be seen that TPS particles were not uniformly dispersed in the PBAT matrix, and large particles were observed, which can be ascribed to the agglomeration of some TPS particles. Between Figure 1d,e, a gradual reduction in domain size and increase in distribution uniformity of TPS particles was observed with increasing glycerol content of the respective compatibilized composites. Among all the composites, the highest improved morphology had the smallest and narrowest distribution of TPS particle sizes in addition to a more uniform distribution of TPS particles, which can be seen in 30% glycerol plasticized PBAT/TPS composite films. It can be explained that 30% glycerol served as the optimum amount of plasticizer content can cause better interfacial bonding between two phases, resulting in finer and improved blend morphology.

### 3.2. Water Absorption

**Figure 2:**
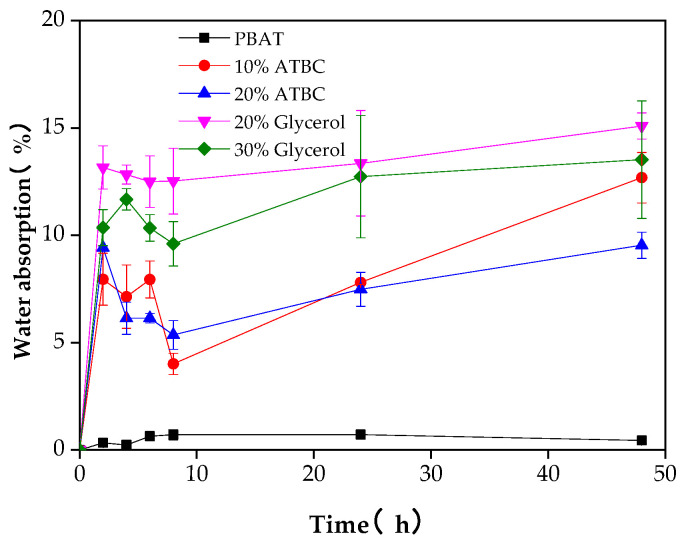
Water absorption of PBAT/TPS films in 48 h.

Water absorption is one of the main shortcomings that restrict the application of starch-based materials. One of the objectives of compounding TPS with hydrophobic polyester is to reduce the water absorption of the film to meet the requirements for maintaining acceptable levels of mechanical properties. The water absorption rates of PBAT/TPS composite films after immersion into distilled water for 48 h are presented in Figure 2, which is consistent with previously published results [19]. Given its hydrophobic nature, the water absorption of PBAT film presented a relatively low water absorption rate (0.44%). The PBAT/TPS composite films presented higher water absorption capacity (maximum 15.09%) after a 48 h incubation period, as compared to the PBAT film, with a significant difference between them [1]. This increase can be attributed to the numerous hydroxyl groups and hydrophilic character of starch. The water absorption of composite films increased rapidly during the first 2 h and then gradually approached the saturation plateau. With 20% ATBC as a plasticizer, the water absorption of the composite films was lower compared to when 10% ATBC was used as the plasticizer, suggesting that increasing the plasticizer content is advantageous for suppressing water absorption. This effect can be attributed to the interaction between the plasticizer and the hydroxyl groups of starch, leading to a weakening of intermolecular hydrogen bonding. A slightly higher water absorption rate was observed in the composite films when glycerol/citric acid/stearic acid were used as plasticizers compared to films with ATBC as the plasticizer. The full continuity in TPS allowed water to access the completely hydrophilic TPS phase through diffusion from the surface via network paths of the TPS phase [1]. Moreover, the incorporation of glycerol introduced more hydrophilic hydroxyl groups during interaction with starch, whereas ATBC introduced ester bonds with stronger hydrophobic characteristics.

### 3.3. X-ray Diffraction Analysis of Films

**Figure 3:**
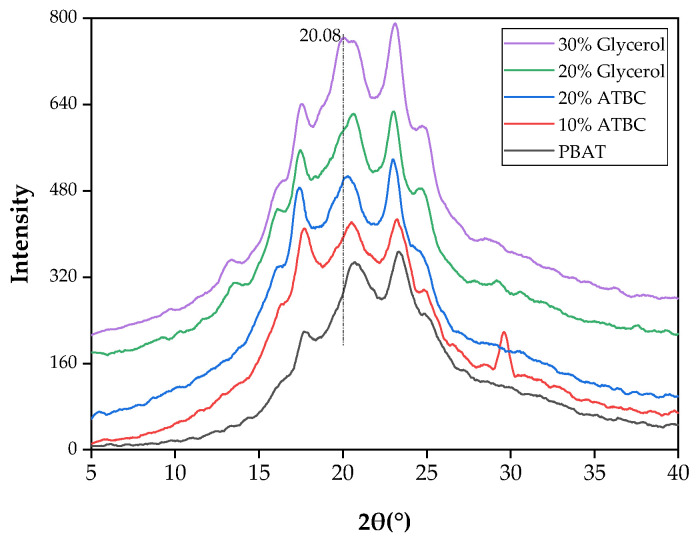
XRD patterns of PBAT/TPS films.

X-ray diffraction patterns of neat PBAT and PBAT/TPS composite films are shown in Figure 3. Peaks at 2θ angles of 17.54°, 20.64°, and 23.14°are characteristic of the crystalline phase of PBAT [20,21]. The starch granule (which presents a certain degree of molecular organization) is partially crystalline and has a degree of crystallinity ranging from 20% to 45% [22]. The corn starch has A-type crystallinity and diffraction peaks in 2θ = 15° [23]. With the addition of glycerol-plasticized TPS, new characteristic peaks appeared at 2θ angles of 13.1° and 16.1° and were characteristic peaks of TPS, which correspond to the V_H_-type amylose crystal [24,25]. The diffraction peaks at 13.38°, 16.24°, 17.54°, 20.64°, 23.14°, and 24.72° were seen in all PBAT/TPS composites, indicating that all composites had a similar crystalline structure. A minor diffraction peak at 20.08 emerged in the 30% glycerol-plasticized PBAT/TPS film. This occurrence may be attributed to the rapid recrystallization of starch during processing, resulting in the formation of a single helical structure complexed with glycerol [26]. It was reported that the transesterification reaction that occurs widely in PBAT/PBS composites not only improved the interfacial compatibility of the composites but also changed their original crystallization types [27]. PBAT/TPS composite films plasticized with 10% ATBC and 20% glycerol showed peaks at 2θ = 29.58°, attributed mainly to the starch retrodegradation during the preservation period [28].

### 3.4. Tensile Property of Films

As shown in Table 1, neat PBAT exhibited a tensile strength of 21.57 MPa and elongation at break of 561.08%, demonstrating high flexibility and ductility. In contrast to the mechanical properties of pure PBAT, PBAT composites with plasticized starch exhibited distinct and varied intrinsic results. The incorporation of ATBC did not enhance the toughness of PBAT when combined with TPS. Table 1 shows that all PBAT/TPS composites not only exhibited more brittle fracture but also demonstrated reduced strength (less than 9.34 MPa), even with an increase in ATBC concentration. The phenomena can be attributed to the compatibility and interface interaction between the polymer matrix and starch [17,29]. An additional factor was the insufficient plasticization process for the crystal structure of starch, which contributed to the poor compatibility between PBAT and TPS. TPS tended to agglomerate in the PBAT matrix, creating stress concentration points when subjected to external force, thereby comprising the mechanical properties of composites. After adding 30% glycerol as the plasticizer, the tensile strength of PBAT/TPS decreased to 17.41 MPa, and the elongation at break decreased to 493.25%, showing a negative impact on tensile properties. The smaller particle size and more regular surface morphology observed in the films plasticized by 30% glycerol aligned with the findings in morphological analyses. This allowed glycerol to penetrate more easily into the molecular structure of PBAT, enhancing compatibility with PBAT. However, the insertion of a small molecule plasticizer and starch between the molecular chains of PBAT weakened the attraction between the molecular chains. The weak intermolecular binding force resulted in the loose crystallization zone of PBAT, thus reducing the strength of PBAT films [17].

After incubating the films at ambient temperature for 48 months, the reassessment of the mechanical properties revealed general decline trends in both tensile strength and elongation at break for all films. The mechanical performance of PBAT composite films containing TPS exhibited a reduction exceeding 50%. However, the elongation at break of the PBAT/TPS composite film plasticized with 30% glycerol still exceeded 400%, possibly due to glycerol migration from the TPS to the PBAT continuous phase [30]. In addition, this indicates that PBAT/TPS composite films are prone to starch retrogradation and degradation during storage. It is crucial to balance the shelf life and biodegradability in practical application.

### 3.5. Thermal Analysis of Films

**Figure 4:**
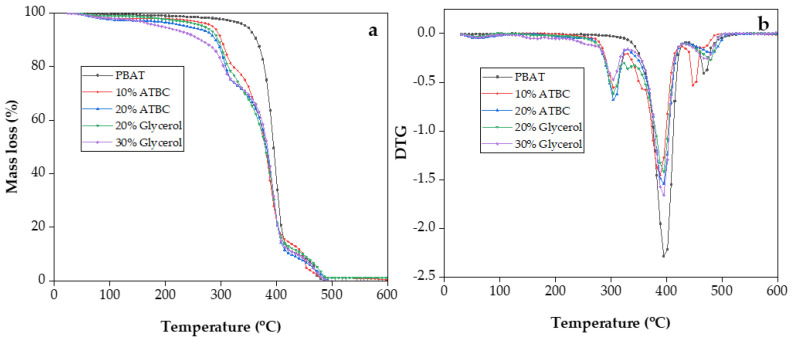
TGA (**a**) and DTG (**b**) curves of PBAT/TPS films during thermal decomposition.

The weight loss curve and DTG curve of PBAT/TPS composite films are shown in Figure 4, and characteristic decomposition temperatures are summarized in Table 2. For all the composite films, minor mass losses were observed in the temperature range of 60–160 °C due to the evaporation of moisture or low molecular weight compounds (Figure 4b). The initial decomposition temperature (T_onset_) was defined as the temperature at which a 5% weight loss of the initial mass occurs (Figure 4a and Table 2). T_onset_ of plasticized PBAT/TPS composite films was lower than that of neat PBAT films. The initial decomposition temperature systematically shifted to a lower temperature with an increase in plasticizer content, which may be attributed to the volatilization of ATBC and glycerol [31]. The degradation stage occurred at a temperature range of 284–340 °C, representing the major decomposition of starch [32]. Further heating at a temperature range of 350–415 °C induced the highest thermal decomposition rate of PBAT, which was reflected by the drastic weight reduction. This stage was ascribed to the elimination of hydrogen groups, decomposition, and depolymerization of the starch and PBAT carbon chains. The DTG peak of 10% ATBC plasticized PBAT/TPS composite films in the temperature range of 430–500 °C shifted to a lower temperature range, indicating the decreased thermal stability of the benzene ring in PBAT compared to other composite films. The final thermal event at 492 °C suggests that all the films decomposed before 500 °C.

In summary, all PBAT/TPS composite films demonstrated a negative impact on the initial thermal decomposition temperature compared to PBAT. This can be attributed to the lower vaporization and decomposition temperatures of the plasticizers. It is noteworthy that in PBAT/TPS films with 10% ATBC as a plasticizer, the rapid decomposition temperature range of PBAT shifted towards lower temperature regions. This shift may be because ATBC weakened the intermolecular forces of PBAT, leading to less tightly packed stacking and, consequently, fewer crystalline regions in both starch and PBAT.

### 3.6. Effect of TPS on Haze and Transparency Property of Films

**Figure 5:**
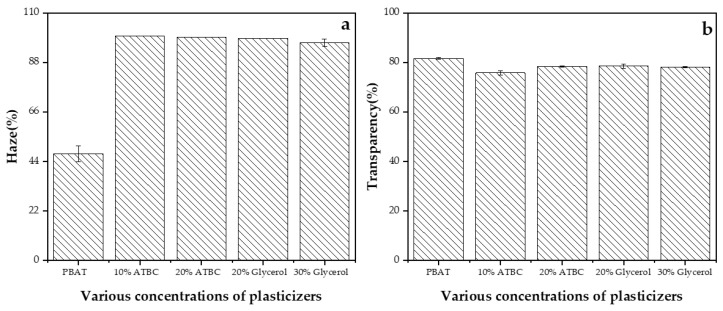
Haze (**a**) and transparency (**b**) of the PBAT/TPS composite films.

The optical properties of films are crucial factors that influence the aesthetics of the films and the purchasing experience for consumers [33]. Figure 5 shows the haze(H) and transparency (T) of PBAT and PBAT/TPS composite films. PBAT/TPS composite films exhibited a slightly lower transparency compared to the PBAT film, particularly evident in films plasticized with 10% ATBC, where T decreased by 7%. This aligns with the findings in haze properties, as all PBAT/TPS composite films exhibited a haze value exceeding 96%, while the haze value for pure PBAT was 47.42%. The inclusion of TPS led to higher haze and opacity and better absorption of visible light (the haze of PBAT/TPS films increased by approximately 50% (*p* < 0.05)) in comparison to the PBAT film. The pore structure of plasticized starch contributed to a larger specific surface area and an irregular microstructure, resulting in an increased refractive index of light and decreased transparency. If the packaged food contains a large amount of lipids, and opacity is desired to minimize light-catalyzed oxidation reactions, the PBAT/TPS composite films would be more suitable than the PBAT film.

### 3.7. Color Variation in Films

Color parameters (L*, a*, b*) of blown films are described in Table 3. The incorporation of 10% ATBC, 20% ATBC, and 20% glycerol as plasticizers slightly increased the luminosity index of the PBAT film, except for the PBAT/TPS films plasticized with 30% glycerol. The brownish color of the starch contributed to heightened redness (a*) and yellowing (b*) in films containing ATBC [34]. Color shifts toward brown and yellow during the melting process may occur due to the loss of structure and crystallinity of starch granules. Samples with lower lightness (L* = 48.88) and comparable yellowing to the PBAT film were observed in the 30% glycerol plasticized films. Elevated amounts of glycerol resulted in films with a darker color, which can be explained by its action as a plasticizer, allowing higher molecular displacement and a less rigid structure. The color analysis complements the UV–vis results, indicating that the chemical constitution of the incorporated starch directly affected the color properties of the developed polymeric films.

### 3.8. UV-vis Transmittance of Films

**Figure 6:**
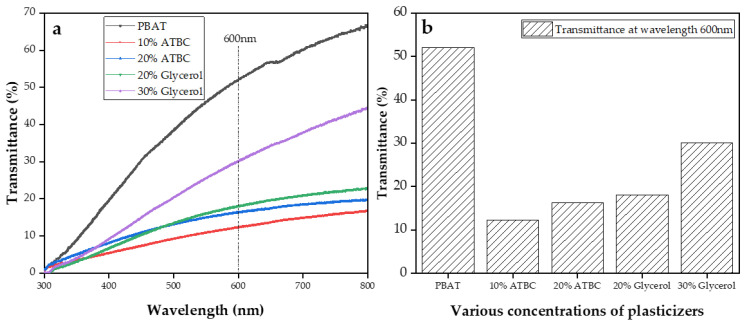
Transmittance of PBAT and PBAT/TPS composite films (**a**), transmittance of films at wavelength 600 nm (**b**).

To further investigate the impact of plasticizers on the transparency of PBAT/TPS composite films, the UV-visible light transmittance of PBAT and PBAT/TPS composite films was measured, as shown in Figure 6. Above 500 nm, transmittance increased with the plasticizer content in the films for both ATBC and glycerol, which was attributed to improved gelatinization. The transmittance of PBAT/TPS composite films, plasticized with 20% and 30% glycerol, exceeded that of films containing 10% and 20% ATBC. The transmittance of light in polymeric films is not only associated with their chemical structure and molar mass but also and, most importantly, dependent on film morphology [35,36]. The morphology of films with starch was affected by the presence of starches that were not completely gelatinized [34]. These particles, which are not fully molten during extrusion, are responsible for refracting most of the light. Films plasticized with 10% ATBC, 20% ATBC, and 20% glycerol exhibited lower transmittance values in the visible light region, likely due to a higher concentration of starch granules not completely gelatinized, as observed in the micrographs. The increased transmittance of films plasticized with 30% glycerol further proved their superior plasticizing effect compared to other PBAT/TPS composite films. The manufacture of biodegradable films with a higher optical barrier is important for most packaging applications [37].

## 4. Conclusions

This study investigated the properties of PBAT/TPS composite films, including starch plasticized using ATBC (10%, 20%) and glycerol (20%, 30%). The films exhibited improved finer morphology, particularly at 30% glycerol concentrations. However, the water absorption of the composite films plasticized with glycerol surpassed that observed with ATBC as a plasticizer; this result can be attributed to the abundant hydroxyl groups in glycerol and full continuity in TPS. The addition of 30% glycerol as the plasticizer led to a decrease in the tensile strength of PBAT/TPS to 17.41 MPa, and the elongation at break reduced to 493.25%, indicating a negative impact on the tensile properties. All PBAT/TPS composite films showed a negative impact on the initial thermal decomposition temperature compared to PBAT, which is attributed to the lower vaporization and decomposition temperatures of the plasticizers. The inclusion of TPS led to higher haze and opacity, demonstrating better absorption of visible light (the haze of PBAT/TPS films increased by approximately 50% (*p* < 0.05)) compared to the PBAT film. Color analysis aligned with UV–vis results, indicating that the chemical composition of the incorporated starch directly influenced the color properties of the developed polymeric films. Films plasticized with 10% ATBC, 20% ATBC, and 20% glycerol exhibited lower transmittance values in the visible light region, likely due to a higher concentration of starch granules not completely gelatinized, as observed in the micrographs. The increased transmittance of films plasticized with 30% glycerol further confirmed the superior plasticizing effect compared to other PBAT/TPS composite films. The production of biodegradable films with a higher optical barrier is crucial for most packaging applications.

As a result, PBAT/TPS composite films present an important option for the development of environmentally friendly and energy-saving packaging materials at a low cost.

## Figures and Tables

**Table 1:** Mechanical properties of films during 48 months of storage at ambient temperature.

	1 Week		48 Months	
	Tensile Strength (MPa)	Elongation at Break (%)	Tensile Strength (MPa)	Elongation at Break (%)
PBAT	21.57 ± 1.47	561.08 ± 6.73	16.89 ± 2.11	499.72 ± 7.84
10% ATBC	6.81 ± 0.56	451.92 ± 9.26	3.28 ± 0.81	12.87 ± 0.48
20% ATBC	7.29 ± 1.62	465.26 ± 6.78	3.51 ± 0.39	43.23 ± 6.61
20% Glycerol	9.34 ± 1.71	378.27 ± 8.43	2.92 ± 0.41	54.85 ± 7.04
30% Glycerol	17.41 ± 2.69	493.25 ± 5.40	8.59 ± 1.26	433.18 ± 8.43

**Table 2:** Characteristic decomposition temperature (T_onset_ and T_max_) of PBAT and PBAT/TPS composite films.

	T_onset_	T_max1_	T_max2_	T_max3_
PBAT	346	304	395	466.5
10% ATBC	285	304/356	388.5	447/466.5
20% ATBC	239	304	395	479.5
20% Glycerol	268	304/330	395	479.5
30% Glycerol	193.5	304	395	479.5

**Table 3:** Color parameters of extruded films.

	L*	a*	b*
PBAT	50.91 ± 2.34	−1.02 ± 0.03	−8.34 ± 0.32
10% ATBC	54.44 ± 0.37	−0.76 ± 0.08	−4.45 ± 0.26
20% ATBC	54.99 ± 0.71	−0.69 ± 0.09	−4.44 ± 0.17
20% Glycerol	55.27 ± 0.97	−1.24 ± 0.16	−6.62 ± 0.08
30% Glycerol	48.88 ± 0.53	−1.10 ± 0.09	−8.30 ± 0.01

## Data Availability

Data are contained within the article and Supplementary Materials.

## Supplementary Materials

The following supporting information can be downloaded at: https://www.mdpi.com/article/10.3390/polym16030326/s1, Figure S1: Stress and strain curves of PBAT/TPS composites after 48 months of storage.
