# Supplementary figures and images for: Effect of Starch Plasticization on Morphological, Mechanical, Crystalline, Thermal, and Optical Behavior of Poly(butylene adipate-co-terephthalate)/Thermoplastic Starch Composite Films
Source: Polymers (Basel). 2024 Jan 25;16(3):326. doi: 10.3390/polym16030326 (PMC10857009; doi:10.3390/polym16030326)

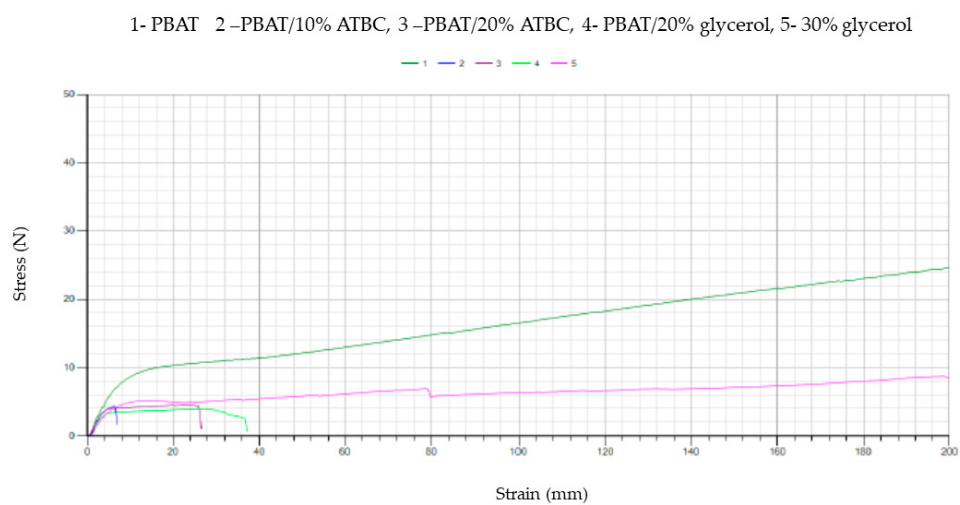

**Figure S1.** Stress and strain curves of PBAT/TPS composites after 48 months of storage.

Supplement: Supplementary file 1 [file polymers-16-00326-s001.zip › polymers-2808320-supplementary.pdf]
